# Therapeutics of Diabetes Mellitus: Focus on Insulin Analogues and Insulin Pumps

**DOI:** 10.1155/2010/178372

**Published:** 2010-05-26

**Authors:** Vasiliki Valla

**Affiliations:** Laboratory of Applied Bioorganic Chemistry, Chemical Engineering Faculty, Aristotle University, 54124 Thessaloniki, Greece

## Abstract

*Aim*. Inadequately controlled diabetes accounts for chronic complications and increases mortality. Its therapeutic management aims in normal HbA1C, prandial and postprandial glucose levels. This review discusses diabetes management focusing on the latest insulin analogues, alternative insulin delivery systems and the artificial pancreas. *Results*. Intensive insulin therapy with multiple daily injections (MDI) allows better imitation of the physiological rhythm of insulin secretion. Longer-acting, basal insulin analogues provide concomitant improvements in safety, efficacy and variability of glycaemic control, followed by low risks of hypoglycaemia. Continuous subcutaneous insulin infusion (CSII) provides long-term glycaemic control especially in type 1 diabetic patients, while reducing hypoglycaemic episodes and glycaemic variability. Continuous subcutaneous glucose monitoring (CGM) systems provide information on postprandial glucose excursions and nocturnal hypo- and/or hyperglycemias. This information enhances treatment options, provides a useful tool for self-monitoring and allows safer achievement of treatment targets. In the absence of a cure-like pancreas or islets transplants, artificial “closed-loop” systems mimicking the pancreatic activity have been also developed. *Conclusions*. Individualized treatment plans for insulin initiation and administration mode are critical in achieving target glycaemic levels. Progress in these fields is expected to facilitate and improve the quality of life of diabetic patients.

## 1. Introduction

Diabetes, being one of the primary causes of increased cardiovascular morbidity and mortality in Western countries, constitutes a large burden to health care systems in terms of both direct and indirect costs. 

 Therefore, efficient glucose control (attainment of normal HbA1C, prandial and postprandial glucose levels) is essential to the prevention of life-threatening complications of the disease. 

 However, most patients fail to sustain long-term, adequate control through life-style modifications or even combined oral medications [1, 2] (biguanides, sulfonylureas, meglitinides, glitazones, incretin mimetics, or DPP-4 inhibitors). Indeed, multidrug therapies are currently reconsidered, with recent studies questioning their long-term therapeutic benefit over side effects or disease complications [3]. 

 Thus, the increasing need of aggressive diabetes treatment has led to the improvement of insulin therapy and its implementation techniques. The development of continuous subcutaneous insulin infusion (CSII) pumps and short-acting insulin analogues exhibiting beneficial pharmacokinetic properties represents important advances in the treatment of diabetes and will be reviewed herein.

## 2. Insulin Therapy

A number of studies confirm that sufficient glycaemic control reduces the risk of developing diabetes-associated complications leading to increased mortality and morbidity. The United Kingdom Prospective Diabetes Study (UKPDS) [4] has shown that a decrease in HbA1C by 1% is associated with 37 and 14% reduction in the risk of microvascular and macrovascular complications, respectively. The link between cardiovascular risk and insufficient glycaemic control has been also confirmed in numerous trials [5, 6], gradually leading to strict guidelines, given the underlying increase mortality and morbidity. 

 Currently, ADA and IDF recommend target HbA1C levels of <7.0 and ≤6.5%, respectively, while the jointed ESC-EASD guidelines recommend that HbA1C target levels should be ≤6.5% in order to reduce cardiovascular risk [7–10].

 Although strict, the official guidelines are poorly met within the diabetic community. In a retrospective analysis of the United Kingdom General Practice Research database [11] published in 2002, 76% of patients were found to have mean HbA1C levels ≥7.0%, while on multidrug therapy including combinations of two or more oral antidiabetic agents (OADs). On the other hand, evidence from lifestyle intervention studies that encouraged weight loss through dietary changes and increased physical activity shows that both the risk of developing diabetes and the progression of the disease may be reduced [12, 13]. However, it is common knowledge that no long-term patient compliance—thus therapeutic efficacy—may be expected. Inevitably, intensive insulin therapy is to be considered for reaching HbA1C targets, as supported by UKPDS, which showed that >60% of type 2 diabetic patients will need insulin within 5 years of diagnosis [4, 14]. 

 Therapeutic algorithms suggest that during early phases of the disease (when the pancreas maintains its secretory ability) should be treated aiming to decrease insulin resistance, then followed by basal insulin. The last therapeutic step is the combination of basal with preprandial insulin to control postprandial glucose fluctuations [14]. 

 Lately, there is a tendency for early initiation of insulin administration but unfortunately, this strategy is held back by the fear of hypoglycaemia, weight gain, and injections [15]. Ideally, insulin therapy restores glycaemic control without interfering with patients' quality of life. This is accomplished by insulin schemes restoring physiological insulin secretion, thus controlling both baseline and postprandial glucose regulation. Therefore, the combinatorial scheme of long-acting basal insulin with rapid-acting analogues is becoming more popular [16]. In any case, it should be taken into account that poorly controlled patients need to manage fasting blood glucose (FBG) levels before postprandial glycaemias.

 Insulin preparations are characterized by the onset of action, peak effect, and duration of action. The current classification includes rapid, short, intermediate, and long-acting products. The insulin's source determines its pharmacokinetic characteristics. 

 The newer insulin analogues have several improvements due to their modified action profile [17]. Main advantages of short-acting preparations include the faster onset of action and shorter duration time. Long-acting analogues afford structural changes, which delay the onset of action, allow slow and continuous absorption into the systemic circulation, and prolong the duration, thus producing a time-concentration profile, which imitates the normal insulin basal level and leads to physiological basal glycaemic control with less nocturnal hypoglycaemias [18].

 In spite of the clinical superiority, the potential mitogenic effect of some analogues remains to be clarified and constitutes a fundamental safety issue. Insulin and IGF-1 receptors display >50% of amino acid sequence homology and even >84% in the tyrosine kinase domain, while both ligands bind to both receptors. On the other hand, insulin molecule modifications in the B10 and B26-B30 region alter the affinity towards the IGF-1 receptor. This has raised the question of mitogenic potential, which may be resulting from an enhanced affinity towards the IGF-1 receptor or because of the occupancy time of the insulin receptor by the analogue. So far, no reliable data on this has been published [19].

Table 1 summarizes the main characteristics of rapid and long-acting insulin analogues described below.

## 3. Rapid-Acting Insulin Analogues

### 3.1. Insulin Lispro

**Figure 1:**
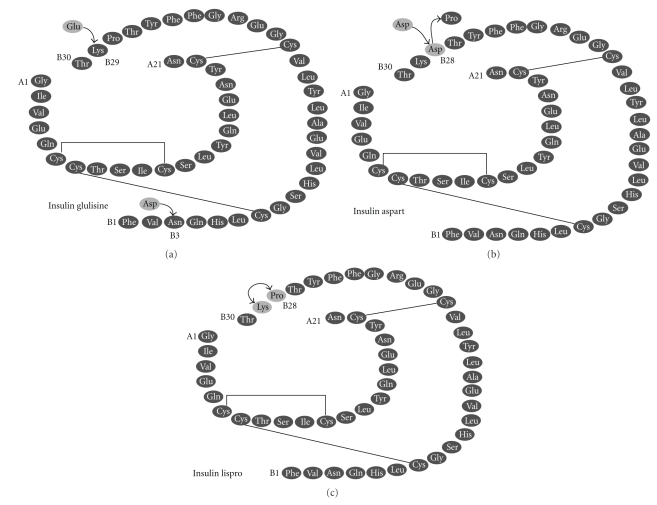
The amino acid structure of rapid-acting insulin analogues. The molecular modifications on the insulin molecule are shown.

Insulin lispro [20, 21] (Humalog) (Figure 1) is the first genetically engineered rapid-acting insulin analogue, approved for clinical use in 1996. Its structure differs from human insulin in the B-chain where proline at position 28 and lysine at position 29 are reversed, leading to a molecule with reduced capacity of self-association in solution (therefore faster absorbed, with higher peak serum levels and shorter action duration in comparison to regular insulin). Besides glycemic management, lispro improves the postprandial leptin and grehlin regulation of type 1 diabetic patients and may be used in cases of gestational diabetes.

### 3.2. Insulin Aspart

Insulin aspart [20, 22] (NovoRapid) (Figure 1) structure differs from human insulin at position 28 where a proline is substituted with the charged aspartic acid, allowing it to be absorbed twice as fast as human insulin. It causes better glycaemic control when administered directly before a meal. Administration of aspart during pregnancy of type 1 diabetic women has been associated with reduced risk of nocturnal hypoglycaemia.

### 3.3. Insulin Glulisine

Insulin glulisine [20, 23, 24] (Apidra) (Figure 1) is the most recent rapid-acting analogue, launched in 2004. Its structure differs in two points from human insulin: asparagine at position 3 is substituted by lysine and lysine at position 29 by glutamic acid. These alterations reduce hexamers formation and enhance absorption from subcutaneous depots. Insulins lispro and glulisine have the same impact on the glycaemic control of type 1 diabetic patients but when evaluating their potential on obese, type 2 diabetic subjects, the rise in insulin concentration and onset of activity is faster for glulisine. In addition, the pharmacokinetic and pharmacodynamic profile of glulisine does not exhibit negative correlation with BMI and subcutaneous fat thickness (which is the case for lispro). 

## 4. Long-Acting Basal Insulin Analogues

### 4.1. Insulin Glargine

**Figure 2:**
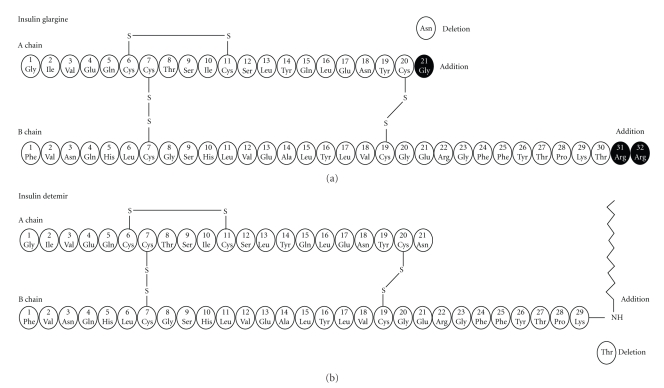
The amino acid structure of insulin glargine and detemir showing the key modifications that result in the prolonged duration of action.

Insulin glargine [25, 26] (Lantus) (Figure 2) is the first long-acting insulin analogue having amino acid modifications in both chains. In the A-chain, the asparagine at position 21 is substituted by glycine and the B-chain is elongated at the C-terminus by addition of two arginine residues. Glargine is a molecule with a changed isoelectric point towards neutral, bearing decreased solubility at physiological pH. This causes precipitation after injection in subcutaneous tissue, stabilization of insulin hexamers, delay of their dissociation, and steady absorption into the circulation. Consequently, insulin glargine bears a stable serum concentration without pronounced peaks and significantly elongated duration of action, which covers the patient for 24 hours. Its onset of action is approximately 2 hours after injection. Compared to NPH or ultralente, glargine affords decreased hypoglycaemic events, less fluctuations, and lower risk of nocturnal hypoglycaemia.

### 4.2. Insulin Detemir

Insulin detemir [25, 27] (Levemir) (Figure 2) is characterized by acylation of myristic acid to the lysine residue at position 29 in the B-chain and deletion of the last threonine (position 30) in the B-chain. Its protracted action is achieved through delayed resorption caused by the increased self-association and reversible albumin binding at the injection site as well as because albumin binding causes buffering of insulin concentration. This results in a flat, prolonged pharmacodynamic profile, which provides a metabolic effect for approximately 17 hours. When compared to NPH and glargine, it offers better weight gain control and results in comparable overall risk of hypoglycaemia.

## 5. Clinical Efficacy and Safety Profile of Rapid and Long-Acting Insulin Analogues

In the Treat-to-Target study [28], insulin glargine was compared to NPH. Both were given at bedtime starting with 10 U, with an aggressive dose titration to achieve a fasting glucose level <100 mg/dL. Both insulins resulted in a substantial decline in fasting glucose and HbA1C, with no difference between groups. Although the FBG target was not achieved in many patients, ~60% of them in both groups achieved their target for HbA1C (<7%) within the 24-week trial period. However, use of glargine was associated with fewer hypoglycemias, including nocturnal ones, in relation to NPH. Similar results have been obtained with insulin detemir when added as basal insulin for type 2 diabetic patients insufficiently controlled with OADs. 

 In the INSIGHT study [29] (implementing New Strategies with Insulin Glargine for Hyperglycaemia Treatment), type 2 diabetic patients (HbA1C levels 7.5–11.0%) on stable therapy with 0–2 OADs, not including thiazolidinediones, were randomized to either an optimised OAD regimen (with no insulin) or initiation of bedtime glargine (with no increase in the oral therapy). The decreases in HbA1C and FPG were higher with glargine compared to OAD therapy (*P* = .005 and *P* = .0001, resp.), with no difference in hypoglycaemia incidences. The INSIGHT study proved that early initiation of insulin glargine improves glycaemic control and is more effective compared to optimised diet/oral therapy alone. 

 The Triple-Therapy Trial [30] compared patients with type 2 diabetes insufficiently controlled with dual oral therapy (metformin-sulfonylurea). Patients were randomised to additionally receive either rosiglitazone or insulin glargine; the doses of which were increased to optimise control. Although similar improvements in HbA1C were observed with glargine (−1.7%) and rosiglitazone (−1.5%), patients receiving rosiglitazone experienced more hypoglycaemias, peripheral oedema, and weight gain. At the end of the study, more patients in the rosiglitazone group had withdrawn. This study demonstrated that single basal insulin administration offers an effective and well-tolerated alternative to further oral drug escalation. 

 Although clearly demonstrated that basal analogues provide satisfactory glycaemic control, their effects on cardiovascular outcomes have been questioned in a recent meta-analysis comparing glargine and/or detemir with NPH [31]. The rates of overall and nocturnal hypoglycaemias were significantly lower in the glargine- and detemir-treated patients but no clinical evidence could support their beneficial effect on mortality, morbidity, or quality of life. However, longer-term studies with glargine or detemir have repeatedly reported on the enhancement of the quality of life and treatment satisfaction. 

 The effects of rapid- and long-acting analogues on HbA1C levels have been thoroughly studied. In a review of 49 randomized clinical studies comparing analogues with regular human insulin in type 1 diabetic patients, a mean difference in HbA1C of −0.1% was assigned in favour of the first [32]. Among type 2 diabetic patients, there was no difference between rapid-acting analogues and regular human insulin. In a similar review of eight studies comparing long-acting analogues with NPH in type 2 diabetic patients, there was no clinically meaningful difference in HbA1C levels between the two types. It should be taken into account though that basal insulin trials are usually designed to titrate dosing as needed to achieve preset HbA1C targets [31].

 A number of studies have also assessed HbA1C levels in patients treated with premixed human insulins versus premixed insulin analogues, including premixed lispro and aspart formulations [32] but the results are contradictory. Only one study showed small improvements in HbA1C levels after treatment with 50/50 premixed insulin lispro relative to premixed human insulins, while the others favoured premixed insulin analogues. In general, patients on premixed insulin analogues exhibit improved postprandial glucose control in comparison to premixed human insulins, probably because of the faster action of the first [33]. 

 The increased risk of hypoglycaemic events remains a major disadvantage of insulin therapy, preventing physicians from applying even more aggressive dosage schemes to lower HbA1C levels. 

 In the DCCT trial [34] (Diabetes Control and Complications Trial), the incidence of severe hypoglycemias was 3-fold higher in the intensive treatment group compared to the conventional treatment cohort (*P* < .001). Moreover, the risk of severe hypoglycemias increased as monthly HbA1C values declined.

 An interesting follow-up of the DCCT has been published. One of the diabetes centres of the initial trial continued to monitor HbA1C levels from 1993 to 1998 for 884 type 1 diabetic patients. During 1993 and 1996, HbA1C continuously declined and that was evidently associated with a significant increase in the number of severe hypoglycemic events (*P* < .001). On 1996, when insulin lispro was launched, 676 patients switched treatment. Surprisingly, HbA1C levels continued to improve (*P* < .001) in the patients switched to lispro, but there was no corresponding increase in the rates of severe hypoglycemia (*P* = .26) [35]. More than that, HbA1C levels did not show further improvement in the subjects who remained on regular insulin. These data suggest that intensive therapy with insulin analogues may not be associated with the same hypoglycemia risks as older formulations. 

 Data on rapid-acting analogues suggest a lower median incidence of severe hypoglycemic episodes per 100 person-years (21.8; range from 0 to 247.4) compared with regular insulin (46.1; range from 0 to 544). Likewise, basal insulin analogue trials exhibit significantly lower risks of nocturnal hypoglycemia with glargine (*P* = .00003) and detemir (*P* < .00001) relative to NPH. The rate of severe hypoglycemia is lower with both basal insulin analogues [36].

 Regarding the critical issue of weight gain, numerous studies have documented that insulin-deriving improvements in glycemic control are frequently accompanied by undesirable increases in body weight [37]. In the DCCT and the Swedish National Diabetes Register trials, modest weight increases were negatively correlated with lipid profiles and systolic blood pressure. Weight gain is highly undesirable especially in type 2 diabetic patients given that more than 80% of them are already overweight. 

 Insulin-mediated weight gain has been attributed to reduced urinary glucose excretion (calorie retention) and to metabolic rate slow down, both resulting from the improved glucose metabolism. The anabolic activity of insulin on both adipose and muscle tissues may be held responsible for prolonged periods of weight gain, even beyond the initial phase of “glucose control-related” weight gain. The rate of weight gain is often greatest during the early months of therapy when glycemic control is also undergoing intensive correction, a parameter, which may interfere with the patient's adjustment to insulin therapy and possibly create an obstacle to his compliance [38].

 For reasons not yet fully understood, some data indicate that insulin detemir has a weight-sparing effect [39]. Trials in patients with type 1 and type 2 diabetes have reported significantly less weight gain in comparison to NPH. While some studies have shown that patients treated with glargine initially gain less weight in comparison to those treated with NPH, no difference between glargine and NPH has been noted in patients treated for 1 year. Moreover, in a recently reported study, patients with type 2 diabetes were switched from NPH or glargine to detemir in combination with oral antidiabetic drugs. Fourteen weeks after the switch to detemir, mean body weight was significantly reduced in patients previously using both NPH (−0.7 kg; *P* < .01) and glargine (−0.5 kg; *P* < .05) [40].

 The impact on quality of life is another factor to be considered when administering insulin therapy. Parameters that may deteriorate quality of life include the patient's concerns about needles, frequent injections, severe hypoglycemias, and weight gain. On the other hand, improved glycemic control itself enhances the patient's state of mind and improves all aspects of everyday activities (including sexual life), therefore motivating treatment compliance [41].

## 6. Continuous Subcutaneous Insulin Infusion (CSII)

The first continuous subcutaneous insulin infusion (CSII) system was developed in 1976 and had the size of a backpack. Initial CSII indications focused on patients who suffered from severe hypoglycemic episodes or hypoglycemic unawareness and type 1 diabetic patients with dawn phenomenon [42]. 

**Figure 3:**
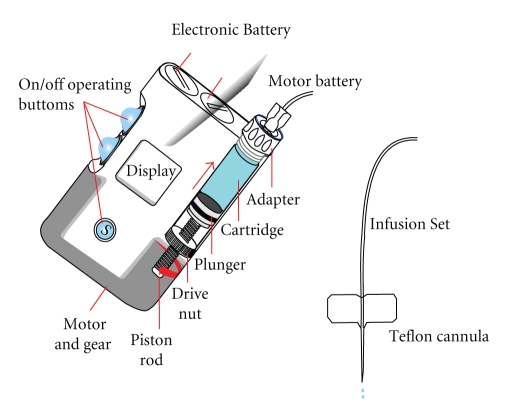
Main parts of an insulin pump.

 CSII is the implantation of an infusion system into the surface of the body for the delivery of insulin. Currently, it is not implanted within the body nor is a closed-loop system. The system (Figure 3) consists of an external device containing a computer, an insulin reservoir containing up to 300 units of rapid-acting insulin, and a screw-drive pumping device. The insulin pump is attached via tubing to a subcutaneously implanted needle (usually in the abdomen) [43]. 

 In more detail, the basic system includes an insulin pump, a plastic needle with a stainless steel stylet for insertion, which is placed in the subcutaneous tissue and plastic tubing connecting a computer reservoir to the implanted needle. In addition, a quick-release mechanism allows the tubing to be disconnected from the implanted needle in the abdomen for temporary pump interruption [44, 45]. Pump components include

- a disposable plastic reservoir that is filled with insulin,
- a screw drive mechanism for “pushing” the insulin through the plastic tubing at different rates,
- a small computer that has a memory loop designed to dispense insulin as programmed,
- a battery source for power.

CSII function is based on the physiologic endogenous insulin secretion [46]. The normal pancreas secretes a varying, basal amount of insulin throughout the day and additional bolus amounts of insulin are secreted to cover the increased prandial needs as a response to the carbohydrates absorption from the gastrointestinal tract. 

 The ideal insulin replacement program should mimic the above endogenous system [47]. The basal secretion is reproduced by the constant slow infusion of rapid-acting analogues. The rate of constant basal infusion can be altered to meet the 24-hour requirements of each individual.

 Bolus injections of rapid-acting analogues are infused via CSII prior to meals or carbohydrate intake, controlling postprandial glycaemias. Candidates must be well motivated and aware of basic insulin pharmacodynamics, carbohydrate counting, and insulin pump technology. Frequent decision-making is required, therefore patients must be able to assess situations and operate a computerized insulin pump. Patients who are not achieving treatment target goals via intensive insulin therapy are also potential candidates [48]. Table 2 summarizes current indications and contradictions of insulin pump therapy.

The total daily dose (TDD) of insulin is calculated based on patient weight or current insulin requirements. If patients are switching to insulin pumps therapy from multiple injections therapy, the TDD is initially reduced by 20%. Alternatively, the initial dose is calculated based on patient weight multiplied by 0.53 IU/Kg (TDD = Wt [kg] × 0.53 U/kg) [49]. The total calculated basal insulin is set on a 1-hourly basal rate, which is adjusted based on fluctuations from the target glucose level. Individual glucose targets are set for different clinical situations (e.g., pregnancy or extensive physical activity) or during different times of the day (e.g., an increase in the basal rate is usually required during dawn hours to offset the rise in counter-regulatory hormones GH and cortisol). The bolus insulin dosage also takes into account the carbohydrate-to-insulin ratio (CIR) [50].

### 6.1. Clinical Studies

Several studies have shown the superiority of CSII over MDI in terms of HbA1C control. Among them, the DCCT Trial [51], in which HbA1C levels in the intensive treatment group were significantly lower with CSII than with MDI (−0.2 to −0.4%). To be noticed though that the patients were allowed to choose between CSII and MDI instead of being randomly allocated. Therefore these results have been questioned.

 Nevertheless, Pickup et al. [52] and Weissberg-Benchell et al. [53], in their meta-analyses including more than 2100 patients, concluded on an overall advantage of CSII over MDI, with a decrease in HbA1C 0.4–0.5% and a corresponding reduction in insulin requirements. 

 Quality of life has been assessed in a limited number of studies, using different parameters and concepts. However, Barnard et al. [54] conclude that CSII has a favourable or neutral effect on quality of life, depression, and anxiety.

 Several randomized controlled trials have shown that CSII with rapid-acting insulin analogues is more efficient for the control of postprandial glycaemia and HbA1C levels than CSII with regular human insulin. Colquitt et al. [55] have demonstrated that the use of insulin analogues with a pump results in a modest (0.26%) but significant reduction in HbA1C compared with soluble insulin.

 The efficacy of CSII compared to MDI therapy has also been evaluated in adapting insulin schemes that combine rapid-acting with NPH insulin as basal insulin [56, 57]. The results suggest that CSII is associated with better glycaemic control, especially in initially suboptimally controlled patients. The relative benefit of CSII over MDI was greater for patients with high baseline HbA1C levels. Nevertheless, the results obtained with CSII were superior to those achieved with MDI, whatever the levels of baseline HbA1C. 

 The 5-Nations trial [58] was a randomized, controlled, crossover trial conducted in 11 European centers, in which 272 patients were treated with CSII or MDI during a 2-month run-in period followed by a 6-month treatment period. The quality of glycaemic control (HbA1C levels), blood glucose values, frequency of hypoglycaemic events, and parameters of life quality were assessed. Hoogma et al. concluded that CSII therapy offers less blood glucose variability and higher quality of life scores.

 Pickup et al. [52] in his meta-analysis assessed CSII versus MDI therapy via the evaluation of 12 randomized, controlled trials and showed a reduction of 0.44 in HbA1C (confidence interval: 0.20–0.69) in patients using CSII. In addition, a reduction of up to 14% in total daily insulin requirements was found for patients using CSII. 

 The impact of basal insulin glargine on CSII therapy has also been investigated. Bolli et al. [59] showed similar glucose control with the two treatment options, while Doyle et al. [60] in a study recruiting children showed the superiority of CSII on HbA1C levels after 16 weeks, with significantly lower average insulin use. In detail, Doyle enrolled 32 conventionally treated subjects who were randomized to either CSII or a combination of insulin glargine and bolus doses of aspart. The CSII group was found to have lower HbA1C levels in comparison to both glargine group and the baseline HbA1C levels. FPG concentrations did not differ between the 2 groups, indicating similar adequacy of basal insulin replacement. However, SMBG performed at lunch, dinner, and bedtime demonstrated significantly lower blood glucose levels in the CSII group, suggesting that lack of compliance with bolus dosing contributes to higher blood glucose and HbA1C levels in the MDI group. In addition, preprandial and bedtime blood glucose values were lower; whereas prebreakfast values were similar, indicating better coverage with CSII. Hirsch et al. [61] in a randomized, crossover study in adults comparing CSII and MDI with insulin reported on lower fructosamine levels and fewer daily glycaemic excursions with CSII, as assessed by continuous glucose monitoring. 

 Most studies point out the fact that CSII superiority depends on patient screening and pre-CSII glycaemic profile. In a retrospective analysis of data from 17 diabetes outpatient clinics in Sweden, Fahlén et al. [62] showed that switching from MDI without long-acting analogues to CSII improved metabolic control, with a more pronounced effect associated with CSII, particularly in patients with higher HbA1C levels and BMI at baseline.

 In all, clinical studies indicate that adult patients, with type 1 diabetes mellitus, are favoured by CSII treatment in comparison to MDI therapy and are led to a better glycaemic control with less hypoglycaemic episodes and insulin requirements [63].

### 6.2. CSII in Paediatric Patients

There is plenty of evidence that both children and adolescents are favoured by CSII equally or more than MDI [64] (Table 3). Cohort studies provide evidence of a significant benefit when paediatric patients on MDI are converted to CSII. 

 For example, Weinzimer et al. [65] found that HbA1C levels continued to fall 4 years after conversion to CSII in young children (mean age 4.5 years; *n* = 65). Sulli and Shashaj [66] have discussed the long-term benefits of CSII observed in older children, with a mean age of 12 years (*n* = 42), followed-up for 4 years after therapy initiation. Recently, Jakisch et al. [67] reported on a prospective study of paediatric patients initiated on CSII or MDI in Germany (*n* = 434 matched pairs). Superior HbA1C control was achieved by CSII during the first year (7.5% versus 7.7% with MDI; *P* < .006) but was not maintained until the third (8.1% versus 8.0%, resp.; *P* < .99). However, CSII was associated with statistically significant reductions in hypoglycaemic and DKA episodes and insulin requirements throughout the follow-up period. Weinzimer et al. [68] randomized almost 300 children and adolescents to 16 weeks of CSII therapy with insulins lispro or aspart. Although a numerically greater reduction in HbA1C with aspart did not reach statistical significance, more patients treated with aspart (59.7%) achieved target HbA1C levels compared with patients treated with lispro (43.8%; *P* < .04).

 Hypoglycaemic episodes constitute an obstacle for many insulin-treated children especially when low glycaemic targets are set. In principle, CSII should reduce the risk of hypoglycaemia. Indeed, Pickup and Sutton [69] in a recent meta-analysis of 21 studies showed that CSII was associated with a 75% lower rate of severe hypoglycaemia compared to MDI. The reduction in hypoglycaemia was the greatest in patients with a high hypoglycaemia rate while on MDI. On the other hand, convenience should be secured to children, for whom MDI administration becomes frustrating when trying to achieve hard glycaemic targets. CSII is more convenient with regard to injection frequency, as the subcutaneous cannula needs to be resited once every 2-3 days. McVean et al. [70] partially attribute the effectiveness of CSII in children to the number of cannula sites used, perhaps due to improved insulin absorption. CSII has been shown to improve the well-being of both children with type 1 diabetes and their parents. Treatment satisfaction is higher among children randomized to CSII than MDI. Recently, Hanas and Ludvigsson [71] detected a small increase in DKA in paediatric patients treated with CSII, as compared with MDI, especially early after CSII initiation. Contradicting that, Jakisch et al. [67] found that a lower baseline incidence of DKA in the CSII group was maintained throughout the 3 years of follow-up of his trial. DKA in pump users is most likely to occur when patients do not realize the malfunctioning of the pump. Thus, proper training and monitoring can minimize the problem. Type 1 diabetic children and adolescents benefit an improved quality of life [72–75] and sufficient glycaemic control when switching to CSII as long as they get accustomed to the mechanical aspects of their treatment.

### 6.3. CSII in Pregnancy

Glycaemic control is crucial during pregnancy for both mother and foetus, because of the close relationship between blood glucose levels and pregnancy outcome [76]. However, achieving stable glucose levels during pregnancy is challenging because of the increased risk of hypoglycaemia due to the passive diffusion of glucose across the placenta and changes of counter-regulatory hormones. In addition, glycaemic control maintenance is difficult because of the continuous insulin requirement alterations, which are decreased in the first trimester and increased in the second half of pregnancy [77].

 The use of CSII during pregnancy dates back to the early 1980s. Initially, CSII was used during the third trimester to prevent macrosomia, but later its use was extended. CSII can be safely started during pregnancy, but it is preferable to start before conception, in order to minimize malformations risk and allow proficiency with the pump. Potential patients should be compliant with capillary glucose testing (8 to 10 times a day) and willing to monitor ketone bodies [78].

 According to ADA guidelines, the goal for FPG before meal should be ≤105 mg/dL, while the 2 hours postprandial plasma glucose should not exceed 130 mg/dl. Currently, insulins lispro and aspart are indicated for administration via CSII in gestational diabetes [79].

 The majority of the studies conducted in pregnant women are observational involving both types 1 and 2 diabetic patient [80]. The number of randomized, controlled trials comparing CSII and MDI is scarce and include a relatively small number of participants. Mukhopadhyay et al. [81] attempted to review the available data and meta-analyzed 6 studies with a total of 213 pregnant diabetic women randomized on CSII or MDI therapy. Data evaluation considered the weighted mean difference and odds ratio for insulin dose, birth weight, gestational age, delivery mode, hypoglycemic episodes, worsening retinopathy, neonatal hypoglycemia, and rates of intrauterine fetal death. Pregnancy outcomes and glycemic control were not significantly different among treatment groups. Yet, a higher number of DKA episodes and diabetic retinopathy were found in the CSII group (not statistically significant). The authors could not conclude on the superiority of one treatment and suggested that large multicenter, randomized, controlled trials are required for this patients group. 

 Although a clear-cut advantage of CSII over MDI during pregnancy does not emerge from the literature, CSII provides greater flexibility in lifestyle, allows an easier control of morning nausea, decreases the number of hypoglycaemic episodes, decreases blood glucose variability, alleviates the dawn phenomenon, copes more easily with delayed gastric emptying, and eases blood glucose control around delivery [82].

## 7. Continuous Subcutaneous Glucose Monitoring (CGM)

The DCCT trial [34] established the correlation of sufficient glucose control with reduced microvascular complications risk. Frequent glucose monitoring was found to be a determining factor for the achievement of glucose control.

 Up until a few years ago, self-monitoring of blood glucose (SMBG) was considered effective for both types of diabetes since it contributed to the improvement of HbA1C and the delay of long-term diabetes complications [83]. However, this technique has suffered some drawbacks. Noninsulin treated diabetic patients show minimum compliance on the grounds of inconvenience and unnecessary pain. On the other hand, measurements scheduling is often poor resulting in useless, incorrectly interpreted information not applicable to an individualized therapy. However, and despite the above difficulties, SMBG is recommended for the management of diabetes in all the major guidelines [84].

On the other hand, continuous glucose monitoring (CGM) provides detailed information regarding blood glucose fluctuations throughout the day, enabling treatment decisions for the diabetic patient. Compared with SMBG, continuous monitoring provides much greater insight into glucose levels throughout the day, enabling the detection of postprandial glucose excursions and nocturnal hypoglycemias or hyperglycemias even in patients whose HbA1C levels suggest a satisfactory blood glucose control [85, 86]. 

 Visualization of the glucose curve over time in relation to meals consumed and exercise assists the patient to comprehend the importance of dosage adjustments. The graphs shown by CGM further enable the patient to self-manage his glucose levels according to his activities and lifestyle. Even more important, the visible data of “on-line” or unblinded systems of CGM help the patient realize the real-time effects of acute treatment decisions as well as their delayed effects [87].

 There are several devices for CGM but only two are usually used. One is the Holter-type of glucose monitoring, where results are shown and analyzed retrospectively; the second uses real-time presentation of glycaemic values. The CGMS of Medtronic (Minimed, Northridge, CA, USA) was the first device approved and remains popular both in USA and Europe. It provides a retrospective graphical view of the glycaemic profile measured during the last three days [88].

 During the years, both invasive and noninvasive CGM techniques have been developed but up to now, only the first have been approved for clinical use. The invasive techniques include the insertion of a subcutaneous glucose sensor that measures interstitial fluid glucose as it osmotically diffuses from the peri-capillary area towards the cells, using either enzymatic (glucose oxidase) or microdialysis technology. Interstitial glucose may lag behind blood glucose by as much as 20 minutes when blood glucose levels are changing rapidly. These devices require calibration using capillary blood determinations. Each sensor continuously measures glucose levels for up to 3–7 days, giving a read-out every 1–10 minutes. The presently available CGM devices provide either historic readouts or real-time reporting. They are also equipped with alarms warning of impending hypo or hyperglycemia [89]. 

 CGM requires thorough education of both the patient and the physician in order to use the readings properly for the adjustment of treatment. Furthermore, the optimal use of CGM requires the ability to perform blood determinations necessary for calibration. It should be noticed, that to date, CGM systems have not been approved for replacement of traditional SMBG devices, which should be used when continuous monitoring results do not reflect the way the patient feels, prior to medication administration or responding to threshold alarms and certainly when the CGM system is being calibrated so as to ensure accurate calculation of glucose readings [90].

 So far, CGM is indicated [91]

- to observe and determine glucose fluctuations: its magnitude, duration, frequency and potential causes,
- to determine the amount of time a patient spends in out-of normal glucose ranges,
- to diagnose and prevent hypoglycemia,
- To determine the impact of lifestyle modification and diet composition on glucose control,

when screening patients for CGM usage, the physician should also take into account some of its current drawbacks [92, 93] and most importantly the:

- inaccuracy of single point measurements compared to SMBG, particularly in hypoglycemic levels,
- delay of glucose measurements,
- disparity of interstitial glucose measurements,
- need for frequent recalibration.

Furthermore, the technique is invasive and patients tend to reject it. The fact that the technology is best in real-time function constitutes another problem because of the intensive education required before being able to make real-time therapeutic decisions. The cost is also a matter to consider given that reimbursement is not available in many countries. 

 In conclusion, CGM technologies continuously improve and tend to be incorporated into everyday practice of diabetic management. Therefore, more interventional trials evaluating the effects of glucose fluctuations on diabetic complications should be conducted to enhance their efficiency and safety.

## 8. Artificial “Closed-Loop” Systems—Artificial Pancreas

Continuous subcutaneous glucose sensing (CGS) is the most recent advance in the control of serum glucose. Real-time continuous glucose sensors have the potential to revolutionize the treatment of diabetes mellitus. 

 A closed loop system monitors glucose levels and supplies insulin accordingly [94]. The ideal closed-loop system, or “artificial pancreas,” should contain three basic elements [95]:

- a safe-delivery device that stores and releases insulin reliably and accurately (insulin pump),
- an accurate, biocompatible glucose-sensing unit potent of frequent or continuous sampling (control algorithm),
- a control system modulating insulin delivery, glucose and maybe glucagon or amylin according to blood glucose levels (continuous monitor).

Additionally, the microcomputer system in an artificial pancreas will sample, filter, and interpret the glucose sensor data, to compare the reading with allowable norms and to accurately control insulin in order to achieve normalized blood glucose levels. This process should operate continuously without mistakes so as to avoid errors leading to severe hypo- or hyperglycemias. This autonomous function is supposed to resemble glucose responsive insulin secretion from the pancreas and should be a fully closed loop approach [96].

 Unfortunately, the accuracy of the current continuous glucose sensors and algorithms is not yet sufficient to permit the loop to be closed. The open loop requires no autonomous control system, as it is the patient who manages insulin delivery on the basis of blood glucose data provided by the sensor. The closed loop, on the other hand, requires a control algorithm to adjust the administered insulin dose according to blood glucose levels [97]. The feasibility of this therapy has been demonstrated in small studies but its performance has not been tested for home use. 

 Currently, two modalities are being developed. 

 The first is an extracorporeal subcutaneous-subcutaneous approach [98] that utilizes subcutaneous glucose monitoring combined with subcutaneously delivered insulin. The minimally invasive nature of this system offers a strong advantage. Its most important drawback lies in the lag time between glucose measurement and insulin delivery, which may be up to 40 minutes followed by the rapid-acting analogue administration, which requires up to 15 minutes for an effect to occur. 

The second is an implantable intravenous–intraperitoneal system [99] in which glucose monitoring occurs intravenously and insulin delivery is performed intraperitoneally. This approach has a greater chance to achieve a fully closed loop system. The delays in the system are shorter than with the subcutaneous-subcutaneous method but it still has longer physiologic delays. With the implantable insulin delivery pump, insulin is introduced into the peritoneum, which is closer to physiologic delivery and absorption.

 There are several obstacles in the development of an artificial pancreas [100, 101], one of which lies in the physiological difference between variations in interstitial glucose and plasma glucose levels. The system has to take into account the time for the sensor to measure the glucose, the time taken by the subcutaneous absorption of insulin and the irreducible time required for insulin action. The total amount creates a certain inertia depending on the speed of changes in plasma glucose concentrations. Another difficulty arises from controlling the rapid, postprandial blood glucose fluctuations. This is why a “mixed” open/closed concept [102] is currently being developed applying hybrid use of the algorithm: autonomous operation (closed loop) during the fasting and interprandial periods and manual, nonautonomous operation of the prandial bolus programmed by the patient according to meal time or composition (open loop).

## 9. Conclusions

The goal of diabetes treatment is to achieve tight glucose control, avoid chronic complications and limit hypoglycemic episodes frequency in everyday life. 

Remarkable improvement has been made on this field with the introduction of insulin analogues. The rapid-acting analogues efficiently control postprandial glucose levels while the long-acting analogues imitate the normal insulin basal level and lead to physiological basal glycaemic control with less nocturnal hypoglycaemias.

 On the other hand, CSII technology has revolutionalized the way insulin therapy is implemented, providing convenient and flexible insulin delivery during routine treatment in children and adolescents improving their quality of life and glycaemic control. 

 However, despite the considerable efforts to improve insulin pharmacokinetics and develop user-friendly monitors and miniaturized insulin pumps, the goal has not yet been achieved, thus research on automated artificial pancreas is in progress. Considerable work remains to be completed before algorithms for the automated regulation of glucose levels become available. 

 Other strategies for diabetes management are also on way. The transplantation of islets of Langerhans was made possible by developing new isolation and immunosuppressant methods. Nevertheless, their rate of success is still low and the number of donors remains insufficient. Insulin secreting cells are created in vitro but they are far from having the same response to glucose as the genuine *β*-cells. 

 Finally, biosystems aiming to mimick natural feedback by combining sensor function (to perceive glucose modification) and effector function (insulin delivery) are also under development in order to improve the everyday life of the diabetic patient. 

 The ultimate challenge on this field remains a device, which would afford

- sensor specificity in a way that the sensor will only respond to glucose variations,
- pharmacokinetics similar to normal pancreatic activity,
- administration comfort by simple long lasting injections (ideally one subcutaneous injection per week or month).

## Figures and Tables

**Table 1:** Main characteristics of rapid and long-acting insulin analogues.

Analogue	Trade name/manufacturer	Onset (min)	Peak (hrs)	Duration (hrs)
Long-acting analogues				
Glargine	Lantus/Sanofi-Aventis	4–6 hrs	No peak	>24 hrs
Detemir	Levemir/Novo Nordisk	4–6 hrs	8–10 hrs	~17 hrs
Rapid-acting analogues				
Lispro	Humalog/Eli Lilly	15–30 min	0.5–2.5 hrs	3–6.5 hrs
Aspart	Novorapid/Novo Nordisk	10–20 min	1–3 hrs	3–5 hrs
Glulisine	Apidra/Sanofi-Aventis	10–15 min	1–1.5 hrs	3–5 hrs

**Table 2:** Indications, limitations and contraindications of CSII in diabetic patients.

**Long-term indications** ⇒* When clear proven benefit over MDI* * is proven and when other intensified* * insulin regimens fail to achieve* * adequate glycaemic control.*	**Short-term indications**	**Contradictions**
(1) Elevated HbA1c with MDI therapy (2) Marked same-day or between-day glucose levels fluctuations (3) Variability of insulin requirements (i) Endogenous causes: dawn phenomenon (ii) Exogenous causes: work type particuliarities (e.g., shift workers or business travellers) (4) Recurrent hypoglycaemia (severe or non-severe) (i) Failure to maintain HbA1c targets (<7.0%) without the occurrence of disabling or frequent symptomatic or asymptomatic hypoglycaemic events (≥4/week) (ii) Incidence of ≥1 episode(s)/year of unexplained severe hypoglycaemia (5) Other reasons: (i) Allergy to insulin (ii) Lipoatrophic diabetes (iii) Very low insulin requirements	** (1) Acute situations** (e.g., in diabetology units): (i) For the treatment of mild ketoacidosis or acute hyperglycaemia (ii) For acute infections (iii) Undergoing enteral alimentation **(2) Transient situations** (i) Severe painful neuropathy (ii) Chronic infections, foot ulcers and all wound-healing cicatrization situations (iii) Pregnancy or the intention to become pregnant	** (1) Absolute Contradictions** (i) Severe psychiatric disorders (ii) Progressive ischaemic or proliferative retinopathy (iii) Due to the patient's environment or pump: (a) A non-educated medical environment (b) Living with extreme circumstances of either heat or cold for professional or personal reasons (c) Underwater diving (d) Exposure to high electromagnetic fields (NMR) **(2) Relative Contradictions** (i) Poor compliance or patient reluctance to live with the current management of treatment, (e.g., frequent visits to diabetology centre, glycaemia monitoring, ketosis testing) (ii) Poor local hygiene and/or *Staphylococcus* presence (iii) In some cases of end-stage renal failure because of acidosis risk attributed to patient incompliance (iv) Sensory or gestural impairment (difficulty with the technical aspects of pump management)

**Table 3:** CSII therapy in pediatric patients.

**Current indications **
Recurrent severe hypoglycemia
Wide fluctuations in blood glucose levels regardless of HbA1c
Suboptimal diabetes control
Microvascular complications and/or risk factors for macrovascular complications
Good metabolic control but insulin regimen that compromises lifestyle (e.g., competitive athletes and needle phobia)
Adolescents with eating disorders
Children and adolescents with a pronounced dawn phenomenon
Ketosis-prone individuals

**Advantages of CSII compared with MDI in children **

Easier and convenient adjustment of bolus insulin dose and nocturnal basal insulin dose
Greater accuracy of insulin dosage
Easier management of infectious illnesses and hyperglycemic episodes
Quality of life improvement
Less injections ⇒ Less anxiety ⇒ better compliance
Flexibility of everyday activities
